# The Use of GIS Technology to Optimize COVID-19 Vaccine Distribution: A Case Study of the City of Warsaw, Poland

**DOI:** 10.3390/ijerph18115636

**Published:** 2021-05-25

**Authors:** Sylwia Krzysztofowicz, Katarzyna Osińska-Skotak

**Affiliations:** Department of Photogrammetry, Remote Sensing and Spatial Information Systems, Faculty of Geodesy and Cartography, Warsaw University of Technology, 00-661 Warsaw, Poland; sylwia.krzysztofowicz@pw.edu.pl

**Keywords:** COVID-19, vaccination scenarios, spatial analysis, Thiessen tessellation

## Abstract

The COVID-19 pandemic is a global challenge, and the key to tackling it is vaccinating a specified percentage of the population to acquire herd immunity. The observed problems with the efficiency of the vaccination campaigns in numerous countries around the world, as well as the approach used at the initial stage of the National Immunization Program in Poland, prompted us to analyse the possibility of using GIS technology to optimize the distribution of vaccines to vaccination sites so as to minimize the period needed to vaccinate individual population groups. The research work was carried out on the example of Warsaw, the capital of Poland and the city with the largest population in the country. The analyses were carried out for the 60–70 and 50–60 age groups, in various approaches and for vaccines of different companies (Moderna, BioNTech, AstraZeneca), used to vaccinate people in Poland. The proposed approach to optimize vaccine distribution uses Thiessen’s tessellation to obtain information on the number of people in a given population group living in the area of each vaccination site, and then to estimate the time needed to vaccinate that group. Compared to the originally used vaccination scenario with limited availability of vaccines, the proposed approach allows practitioners to design fast and efficient distribution scenarios. With the developed methodology, we demonstrated ways to achieve uniform vaccination coverage throughout the city. We anticipate that the proposed approach can be easily automated and broadly applied to various urban settings.

## 1. Introduction

The COVID-19 pandemic has changed the functioning of societies, education [1], science and various sectors of the economy [2,3] around the world, on all continents. Never in history has an epidemiological threat affected so many societies—their health and functioning at the same time. As of 31 March 2021, 128,540,982 cases were confirmed worldwide, and 2,808,308 people died [4]. In Europe, we are dealing with another, third wave of the pandemic. In the second half of March 2021, the daily number of cases per 1 million inhabitants exceeding 1000 people was observed in Estonia, Hungary, San Marino and Serbia. More than 600 cases per 1 million inhabitants were found in Czech Republic, Montenegro, Kosovo, France and Poland [5]. More countries are introducing restrictions and tightening the sanitary regime, including restrictions on the cross-border movement of people, as well as regional or national hard lock down measures. These decisions help to control the pandemic and flatten the COVID-19 incidence curve [6,7,8] but have a negative impact on the economy and increase unemployment.

According to World Bank experts, the COVID-19 pandemic has led to the deepest global recession in eight decades, despite unprecedented policy support [3]. It has affected the vast majority of sectors of the economy. Most countries experienced a visible recession in 2020. In the second quarter of 2020, GDP (Quarterly Growth Rate) in most European Union Member States reached one of the lowest levels (on average −11.2%, but e.g., in United Kingdom as much as −18.8%, and in Spain −17.9%) [9]. Carefully observing the development of the pandemic, it can be assumed that 2021 will also not be favourable for the functioning of many sectors of the economy (e.g., transportation, tourism, gastronomy, various services).

Experts in the field of epidemiology, including the European Center for Disease Prevention and Control (an agency of the European Union) and WHO, indicate that the only way to combat the COVID-19 pandemic is to massively vaccinate humanity around the globe [10,11]. Thanks to this, a so-called herd immunity (also called herd protection) will be acquired. Herd immunity will ensure the safety of both vaccinated people, but also protection against infection for non-immunized people who do not want or cannot be vaccinated (infants, chronically ill people with certain health conditions or with significantly reduced immunity). A high number of people in the population who have acquired immunity (vaccinated persons or those who have already contracted the disease) greatly reduces the likelihood of infection for those who cannot be vaccinated. As mentioned by Omer et al. the herd immunity threshold is defined as the proportion of individuals in a population who, having acquired immunity, can no longer participate in the chain of the virus transmission. In the simplest model, the herd immunity threshold depends on the basic reproduction number (R_0_; the average number of persons infected by an infected person in a fully susceptible population) and is calculated as 1 − 1/R_0_ [12]. Herd immunity threshold is different for different diseases but also different societies, and it changes as the epidemic proceeds, because some people become infected and gain immunity [12,13,14]. The epidemic intensity of COVID-19 is strongly shaped by crowding. It has been found to be greater in big and high-density municipalities compared to smaller cities and rural areas [15,16]. Large cities with high inter- and intra-city mobility flows have more difficulties in containing epidemic spread [16]. In many studies, the mobility of the population has been indicated as a key mechanism of the accelerated infectious disease transmission [17,18]. Reaching herd immunity depends in part on what’s happening in the given population, but also how effective the vaccine is and how long its protection lasts [12,18]. In the case of COVID-19, it is indicated that the safest range for obtaining herd immunity is at least 60–80% [19,20,21]. However, some scientists point out that it may not be obtainable in the case of COVID-19 [14]. The reasons they provide include: the slow vaccine roll-out, emergence of new virus variants, delayed arrival of vaccinations for children. Also, the effectiveness of vaccines is not yet fully known and whether they prevent the transmission of the virus. The COVID-19 pandemic has made the very concept of herd immunity increasingly questioned [14]. Nevertheless, vaccination is the key element in defeating the pandemic. A retrospective study conducted in Israel indicates that the rapid and mass vaccination roll-out resulted in a significant drop in COVID-19 cases (by 77%) and hospitalizations (by 68%). It has been shown that a decrease in these clinical measures depends on the rate of vaccination—the effect was greater in cities where a higher fraction of individuals was vaccinated earlier [22].

**Figure 1:**
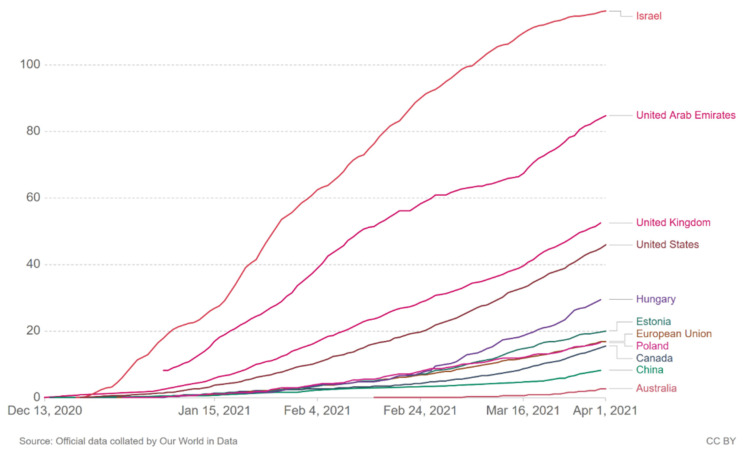
COVID-19 vaccine doses administered per 100 people (1 April 2021) [5]. Total number of vaccination doses administered per 100 people in the total population. This is counted as a single dose and may not equal the total number of people vaccinated, depending on the specific dose regime (e.g., people receive multiple doses).

On 19 January 2021 the European Commission set out actions to step up the response against the COVID-19 pandemic and accelerate the rollout of vaccination campaigns across the EU, with the aim to vaccinate a minimum of 70% of the adult population (over 18 years old) by the summer of 2021 [23]. Such a high threshold of vaccination of the population is a huge logistical challenge, especially taking into account the limited availability of vaccines and the strict conditions of their storage and distribution. All EU Member States have developed own strategies for the deployment of the COVID-19 vaccine at the national level, planning the rollout of their national vaccination campaigns in phases. Phase one, consisting of vaccination of the priority groups, has started in all countries. Priority groups have been selected based on their higher risk of developing severe disease, as well as to protect healthcare and other front-line workers [23]. As of 1 April 2021, 70,684,970 doses of vaccines have been administered in the European Union countries [24]. The first dose was received by 13.4% and the second by 5.6% of the population [24]. As shown in the diagram (Figure 1), the vaccine roll-out is faster in Israel (ca. 60% of the population was vaccinated with at least one dose), the United Arab Emirates, the United Kingdom (ca. 27%) and the United States (ca. 45%) [5]. The European Union is struggling with untimely supply of vaccines by pharmaceutical companies and hence, the vaccination rate in Europe is slower than planned.

The vaccination campaign against COVID-19 in Poland began on 28 December 2020. By 1 April 2021, 6.27 million people have been vaccinated, of which 4.23 million (approx. 13.5% of the adult population) with the first dose, and 2.04 million (approx. 6.5% of the adult population) with two doses [24,25]. In the last several days, as many as 180–190 thousand people are vaccinated daily [26]. In Poland, however, to achieve the herd immunity threshold of 70%, it is necessary to vaccinate approximately 27 million adult inhabitants of the country. Only then will it be possible to return to the normal functioning of society and the economy, which has already suffered huge losses. The sector which has been particularly hard hit is the small and medium-sized enterprises (SME) sector [24,27].

The National Immunization Program in Poland assumes that vaccinations are voluntary and rolled out in phases [28]:

- Phase 0: employees of the health care sector, employees of Residential Care Homes and Municipal Social Services Centres as well as auxiliary and administrative staff in health care facilities, including sanitary and epidemiological stations.
- Phase I: residents of Residential Care Homes, Nursing Homes and other residential care facilities, people over 60 years of age in order of age, uniformed services, teachers.
- Phase II: people under the age of 60 with chronic diseases that increase the risk of severe COVID-19, or during diagnostic testing and treatment, requiring repeated or continuous contact with health care facilities, people directly ensuring the functioning of the basic state operations and at risk of infection due to frequent social contacts.
- Phase III: entrepreneurs and employees of sectors closed under the regulations on establishing specific restrictions, orders and bans in connection with the outbreak of an epidemic, general vaccination of the rest of the adult population.

The head of the Chancellery of the Prime Minister—Michał Dworczyk, at a press conference on 1 February 2021, announced that 94% of medical sector employees had been vaccinated as part of Phase 0 [29]. The data published on the website of the Ministry of Health [25] shows that so far (as of 1 April 2021) 80% of people over 75 years of age have been vaccinated, 64% in the 71–75 age group, 22% in the 61–70 age group, 14% in the 51–60 age group, 12% in the 41–50 age group, 8% in the 31–40 age group and 6% in the 18–30 age group.

In order to achieve the level of herd immunity as quickly as possible, it is important that the vaccines reach individual vaccination sites in the optimal number, taking into account the needs, but also minimizing the risk of spreading the virus in the population. For this purpose, Geographic Information Systems (GIS) technology can be used. This is a broad term referring to a number of methods related to all components of geographic information systems for example data or analysis. In particular it offers a possibility of performing various types of spatial analyses to inform the decision-making process, wherever the spatial aspect is important. This technology is widely used in analyses related to the assessment of the accessibility of various types of natural, semi-natural and anthropogenic objects by inhabitants of cities or other areas [30,31,32,33,34,35]. In the case of the COVID-19 pandemic, this technology was used extensively to understand the spatiotemporal dynamics of COVID-19. The analyses conducted until now have mainly examined spatial distribution and analysis of COVID-19 cases and/or deaths in relation to geographic area, age, sex, health, living conditions, socio-economic and environmental variables [36,37], as well as spatiotemporal COVID-19 patterns and their changes [38,39], early forecasting of the potential risk zones of COVID-19 [40], and risk modelling of COVID-19 infections [21,41,42,43]. However, the use of spatial analysis may also be useful in optimizing the location of vaccination sites and the distribution of vaccines. GIS technology has been used so far in research on vaccination against various infectious diseases. The researchers’ interest has focused on planning vaccination campaigns, identifying populations that have not been vaccinated, and assessing the progress of vaccination campaigns [44,45,46,47,48].

Due to the vaccination model currently adopted in Poland, the inhabitants are often referred for vaccination to distant places, where doses of vaccines become available quicker than near their place of residence. This approach requires travelling longer distances and increases the risk of possible infection. Therefore, there is a clear need for optimizing vaccination distribution in largest regions or even the entire country to ensure faster and administration of vaccines in multiple locations at the same time. The aim of our study was to examine the possibility of using GIS technology (its different tools and methods) to quickly analyse various vaccination scenarios and optimize the delivery of vaccine doses to individual vaccination sites in order to minimize the time needed to vaccinate the population in a specific age group. The analysis covered scenarios with the use of vaccines from different manufacturers, as well as with equal or population-dependent distribution of vaccine doses to each vaccination site.

## 2. Materials and Methods

**Figure 2:**
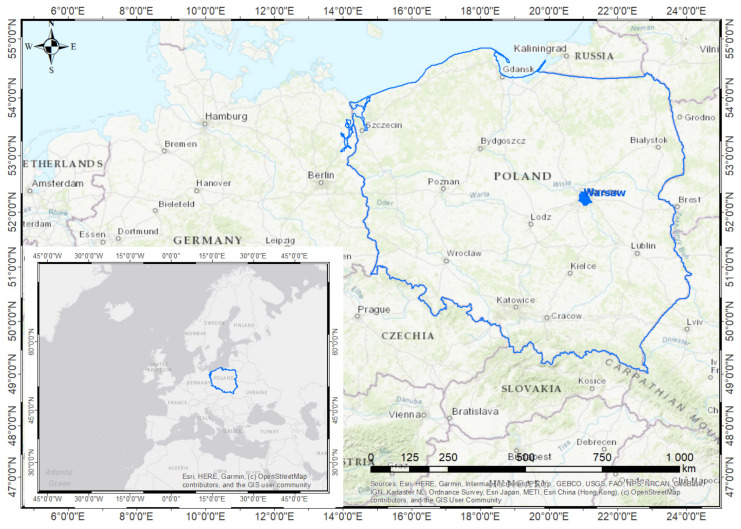
Location of the study area.

**Figure 3:**
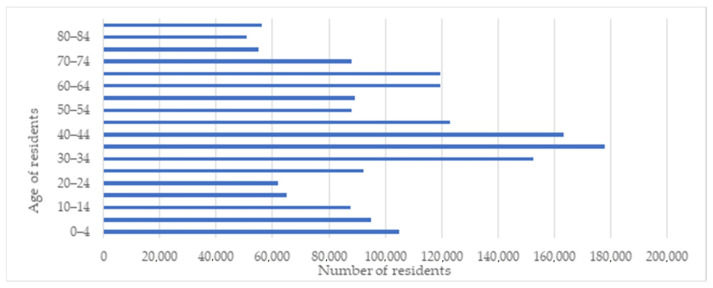
Demographic structure of Warsaw residents.

The research work was carried out for the City of Warsaw—the capital of Poland (Figure 2). It is a city of 1,790,658 inhabitants, the largest in the country, and a population density of 3462 people/km^2^ [49]. Figure 3 shows the demographic structure of the city’s inhabitants [50]. Adults aged 18 years and older represent 81.7% of all city residents. The group of people showing the greatest mobility and activity levels, i.e., people aged 18–44, constitute 37.6% of the population. People over 44 years of age are the most numerous group, which accounts for 44.1% of all city’s inhabitants. The smallest group are children and adolescents—people under the age of 18 constitute 18.3%.

Until now (as of 1 April 2021) 25% of Warsaw inhabitants have been vaccinated, with two doses of vaccine administered to 8.3% of the population [24,49]. Recently, 8–11 thousand people have been vaccinated daily [24]. In order to reach the vaccination threshold assumed by the EU, a minimum of 70% (1,024,020) of adult residents of Warsaw, i.e., 787,000 people, still need to be vaccinated by the summer of 2021. This means that over 8500 residents of Warsaw should be vaccinated daily. Taking into account the high mobility of the inhabitants of large cities and the high population density, a greater percentage of the population should probably be vaccinated in order to achieve herd immunity.

### 2.1. Materials

The source data for this study were: data from the personal identification number (PESEL) register aggregated to the building level and the location of COVID-19 vaccination sites in the Mazowieckie voivodship.

The PESEL register is the central data set on the population kept by the ministry responsible for digitisation in Poland [51]. The register stores data on all Polish citizens living in Poland and abroad, as well as on foreigners in connection with the procedure of applying for the right of permanent residence or other right to reside in Poland. The register includes detailed data identifying a person, including name and surname, date of birth, place of birth, gender, permanent residence address, marital status. Data in the register are updated on an ongoing basis, and access to the register is granted to specific public administration bodies which need it to perform the tasks assigned to them specifically under the law. In other cases, the ministry of digitisation provides aggregated data from the register for a specific moment in time under the procedure of access to public information.

Data from the register were collected for the age groups analysed in this study, i.e., people aged 50–60 and 60–70. These are the groups that according to the National Immunization Program in Poland are either currently being vaccinated or their vaccination is to begin soon. Data aggregated to the level of a single address/building, in which at least one person is registered, were obtained, including:

- number of people registered in the building,
- number of apartments in which at least one person is registered,
- number of people broken down by gender and five-year age groups, e.g., the number of women aged 50–55 registered in the building, the number of men aged 50–55 registered in the building, the number of women aged 55–60 registered in the building etc.

The address data was geocoded, which made it possible to create a spatial data set containing information about the demographic structure of people registered as residents of Warsaw. The data are valid for 16 June 2020. In Poland, in the case of persons aged 50 and above, one can observe a much lower mobility in terms of their actual place of residence differing from their place of registered residence, which is caused by many factors, from economic to those related to health and individual preferences [52]. Therefore, in this study it was assumed that the demographic structure of the city’s registered residents aged 50 and over is the same as that of the actual residents in this age group, which enabled identification of areas with a high density of residents aged 50 and over.

Data on the location of COVID-19 vaccination sites were obtained from the Mazowieckie Voivodeship branch of the National Health Fund (NFZ). The data were shared on a map portal: https://mownfz.maps.arcgis.com/ (accessed on 25 May 2021). Each vaccination site is described by the following set of characteristics:

- name of the site/facility where vaccination is carried out,
- number of the entry in the Register of Entities Performing Medical Activities,
- information on whether primary healthcare services are provided at a given site,
- contact details of the vaccination site,
- geographic coordinates.

**Figure 4:**
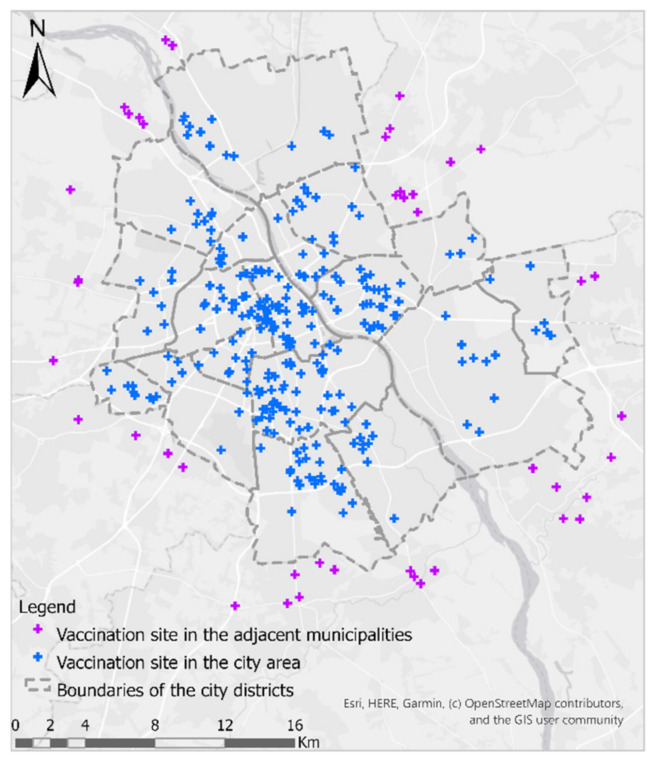
Location of COVID-19 vaccination sites in the area of the Capital City of Warsaw and at a distance of 3 km from its borders.

The data are valid for 15 February 2021. This study uses data on the location of vaccination sites in the city and those located within 3 km from the Warsaw borders (Figure 4). It includes 268 sites within the city, and 44 in neighbouring municipalities.

### 2.2. Methods

The availability of COVID-19 vaccination sites was analysed with the use of GIS technology, which makes it possible to take into account the distribution of sites in relation to areas inhabited by people of a specific age group. The applied approach allowed to identify problem areas where the number of vaccination sites is too small in relation to the number of people from the currently vaccinated age groups. It was also possible to simulate the number of days needed to vaccinate people of specified age groups, taking into account different vaccination scenarios and recommendations for intervals between doses of vaccines.

**Figure 5:**
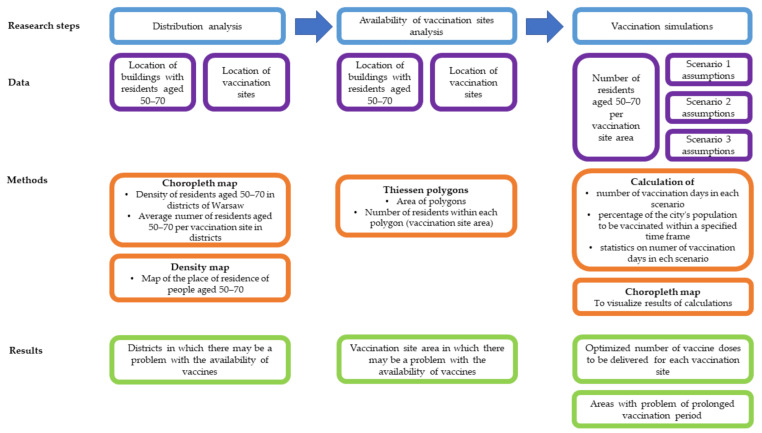
Methodology scheme.

The research consisted of the following steps (Figure 5):

(1) analysis of the distribution of buildings inhabited by people over the age of 50 and COVID-19 vaccination sites in the area of Warsaw,
(2) analysis of the availability of currently operating vaccination sites (as of 1 April 2021) located in Warsaw,
(3) vaccination simulations according to various scenarios—determining the number of days needed to vaccinate different age groups.

#### 2.2.1. Analysis of the Distribution of Buildings Inhabited by People over the Age of 50 and Vaccination Sites in the Area of Warsaw

The analysis of the distribution of areas inhabited by people over 50 and of vaccination sites was made using one of the cartographic data presentation methods—the choropleth map. It is a quantitative method that enables a visualization of the numerical features of a given phenomenon in relation to a defined surface—the base field [53]. Choropleth maps were made using ArcGIS Pro software (ESRI, Redlands, USA). With its help, the following visualizations were made: population density of people over 50, number of vaccination sites per 10 km^2^, number of people over 50 per one vaccination site, with the districts of Warsaw adopted as the base fields. This method of analysis enabled a visual assessment of the presented phenomenon and drawing quick and accurate conclusions about districts where the number of vaccination sites is too low in relation to the number of inhabitants 50+.

In the analysis of the distribution of places inhabited by people over 50, the method of converting discrete data, i.e., points representing buildings, into continuous data, i.e., a raster showing the population density of people over 50, was also used. For this purpose, kernel function was used, specifically the quartic kernel function [54]. The density of buildings weighted by the number of residents in a 1000-m neighbourhood was calculated according to the following Equation (1):

(1) $$Density=\frac{1}{r^{2}}\overset{n}{\underset{i=1}{\sum }}\left[\frac{3}{\pi }\cdot w_{i}{\left(1-{\left(\frac{d_{i}}{r}\right)}^{2}\right)}^{2}\right],\;for\;d_{i}<r$$

where *i* = 1,…, n—the input points, *r*—radius distance, *w_i_*—the weight field value of point I, *d_i_*—distance between point *i* and the x, y location (centre of the raster cell).

The applied method allowed a visual assessment in a continuous manner of the distribution of places inhabited by people over 50 within the city area without relating it to the administrative division.

#### 2.2.2. Analysis of the Availability of Vaccination Sites Located in Warsaw

The analysis of the availability of vaccination sites within the city was performed using the Thiessen polygons method, which divides a given area in such a way that each point located within a given polygon has the shortest distance to the node appropriate for this polygon, compared to the other nodes. In order to create Thiessen polygons, an algorithm implemented in the ArcGIS Pro software was used, which consists of two stages:

(1) triangulation of input points (nodes) and creation of a TIN (triangulated irregular network) that meets the Delaunay criterion,
(2) polygons creation by joining the intersections of bisectors of each side of the triangle belonging to the TIN.

The input data to the algorithm were the COVID-19 vaccination sites. In the case of this vaccination, there is no so-called “regionalization”. This means that people from the currently vaccinated group can sign up for vaccination at any vaccination site. However, taking into account the state of pandemic, and thus the movement restrictions, which may also result from the age of the analysed age groups, it was assumed that the optimal vaccination site is the one located closest to a person’s place of residence. Taking this into account, the number of residents inhabiting a polygon area was added to each Thiessen polygon. The results were presented by a choropleth map, which enabled identification of vaccination sites, in the case of which the assigned number of people is so high as to indicate problems with a prolonged time needed to vaccinate the studied population. The Thiessen polygons allow conclusions to be drawn both in relation to the even distribution of vaccination sites in the city area and their accessibility.

#### 2.2.3. Simulations of the Number of Days Needed to Vaccinate Each Studied Age Group

Based on the population assigned to the vaccination site and assuming a specific number of vaccines per site, the number of days needed to vaccinate the specific age groups was calculated. The analysis covered the age groups for which vaccination is to begin in Poland in the near future—people aged 60–70 and people aged 50–60. The simulations were carried out in various scenarios.

*Scenario 1*

**Figure 6:**
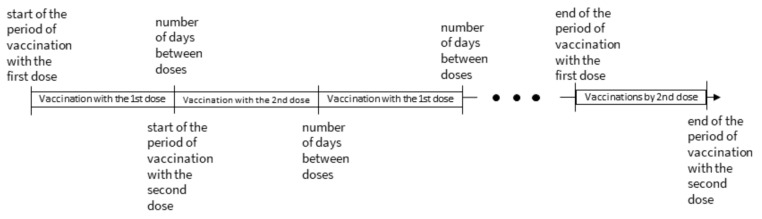
Assumed vaccination scheme with two-dose products.

Even distribution of vaccines across all vaccination sites, assuming the number of doses delivered and administered each day is 30 (the scenario implemented in the first weeks of the vaccination campaign in Poland). Vaccinations were performed with two-dose vaccines, which are currently administered to Polish citizens, i.e., of Pfizer/BioNTech, Moderna and AstraZeneca. For the mRNA vaccines the current interval between doses recommended and used in the National Immunization Program in Poland is a maximum of 42 days [55], and for Astra Zeneca vaccine it is 84 days [55], reduced by one day. The number of days needed to vaccinate all persons at a given vaccination site was determined assuming alternating vaccinations according to the scheme presented in Figure 6.

*Scenario 2*

Vaccine distribution optimized in terms of the number of inhabitants assigned to a given vaccination site, assuming the maximum daily number of vaccinations is 132 and the same total number of vaccines per Warsaw as in scenario 1. The assumed maximum number of people who can be vaccinated is based on the assumption of a vaccination site being open for 11 h and vaccinating one person every 5 min. The intervals between doses and the vaccination scheme were adopted in the same way as in scenario 1.

*Scenario 3*

Vaccinations are carried out with a single-dose vaccine with even distribution between vaccination sites amounting to 30 pieces (a) and optimized in terms of the number of inhabitants (b). This scenario was presented in terms of the possibility of introducing the Johnson & Johnson product in Poland, which, according to media reports, is to be available in the second quarter of 2021. The number of days needed to vaccinate a specific group of people was determined with the following Equation (2):

(2) $$D=[\frac{pop}{d}]$$

where *D*—the number of days it takes to vaccinate a certain number of people, *pop*—number of people to be vaccinated at a given vaccination site and *d*—daily number of delivered vaccines.

The proposed method of optimizing the number of vaccines delivered to vaccination sites was carried out in the following steps:

(1) calculating the median number of days needed to vaccinate the number of people assigned to a given vaccination site, assuming an even distribution of the vaccines (*M_D_*),
(2) calculating the number of doses according to the following Equations (3) and (4):
  (3) $$for\;\frac{pop}{M_{D}}\geq max_{d}\;\underset{}{\Rightarrow }\;d_{1}=max_{d}$$
  (4) $$for\;\frac{pop}{M_{D}}<max_{d}\;\underset{}{\Rightarrow }\;d_{1}=[\frac{pop}{M_{D}}]$$
  where *pop*—number of people to be vaccinated at a given vaccination site and *max_d_*—the maximum daily number of vaccines that can be distributed at one vaccination site.
(3) scaling the number of doses determined in step 2 with a factor (*c*):
  (5) $$for\;d_{1}=max_{d}\;\underset{}{\Rightarrow }\;d=max_{d}$$
  (6) $$for\;d\neq max_{d}\;\underset{}{\Rightarrow }\;d=[c\cdot d_{1}],\;c=\frac{\sum_{i=1}^{n}d_{ui}}{\sum_{i=1}^{n}d_{1i}}\;$$
  where *i* = 1,…, *n*—number of vaccination sites, *d_u_*—the daily number of doses, assuming an even distribution of vaccines between vaccination sites and *d*—optimized daily number of vaccine doses to be assigned and delivered to a given vaccination site.

A description of the simulations carried out in this study is provided in Table 1, together with the abbreviated names that will be used for the simulations throughout the text.

For all simulations, the following statistics were computed and used to compare the scenarios: the mean value, quantiles values, quartiles values and max values of the number of days needed to vaccinate a specific age group at all vaccination sites in Warsaw. Finally, the percentages of the city’s population which would be vaccinated within a specified time frame were determined for the three vaccine distribution scenarios.

## 3. Results

### 3.1. Analysis of the Distribution of Buildings Inhabited by People over the Age of 50 and Vaccination Sites in the Area of Warsaw

**Figure 7:**
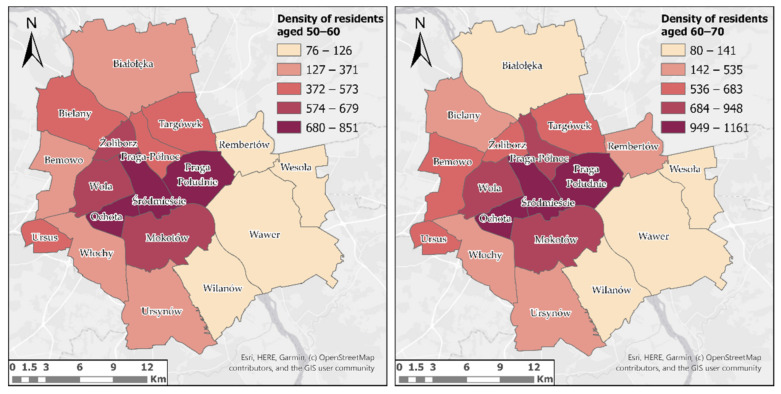
The results of the analysis of the density of the older population by Warsaw districts.

Preliminary analyses of the population density of residents aged 50 and over showed that it is the highest in central city districts, and the lowest in peripheral districts (Figure 7). 

In the case of the most densely populated districts, both in the 50–60 and 60–70 age groups, these are Ochota, Śródmieście and Praga Południe. As for the districts with the lowest density of older adults, for both age groups these are Wilanów and Wawer. It should also be noted that the total number of people in the 60–70 age group is slightly higher than the number of people in the 50–60 age group.

**Figure 8:**
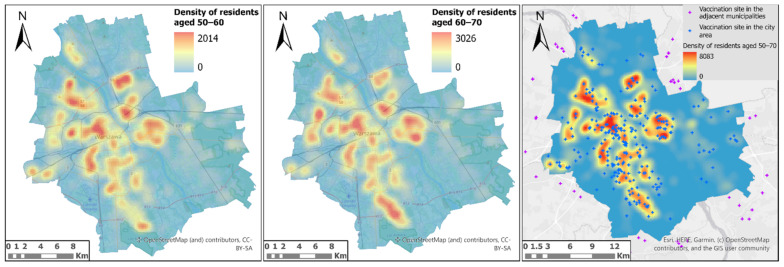
The distribution of hot spots of high population density in analysed age groups.

When analysing the results of the population density without reference to the district division, it is easy to notice that the distribution of hot spots of high population density in both analysed age groups is very similar (Figure 8). Large clusters of residents over 50 are found in places where large housing estates are located along or near the main communication routes of the city. A preliminary visual analysis of the distribution of COVID-19 vaccination sites in relation to hotspots with high population density appears to be optimal. The number of vaccination sites is significantly higher in the central parts of the city and lower in the peripheral areas, which is in line with the results of the analysis of housing density.

**Figure 9:**
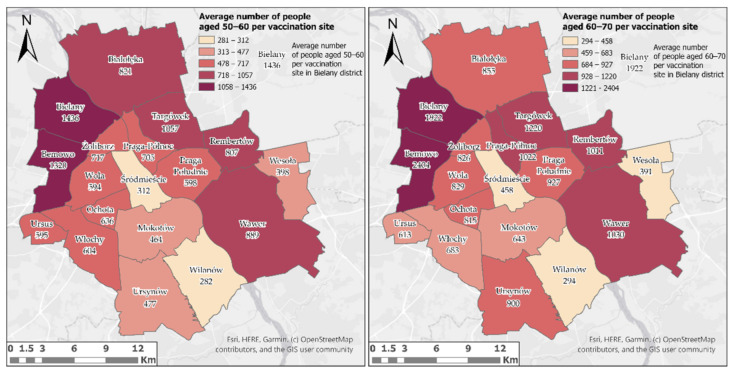
Average number of people in both analysed age groups per vaccination site in districts of Warsaw.

However, taking into account the average number of people per vaccination site in the district, it is possible to clearly identify districts in which there may be a problem with the availability of vaccines (Figure 9). A large number of people per vaccination site may indicate an extended period of time needed for administration of vaccines. Unfortunately, for both analysed age groups this problem occurs in the same districts—Bielany and Bemowo, as shown in Figure 9.

### 3.2. Analysis of the Availability of Vaccination Sites Located in Warsaw

**Figure 10:**
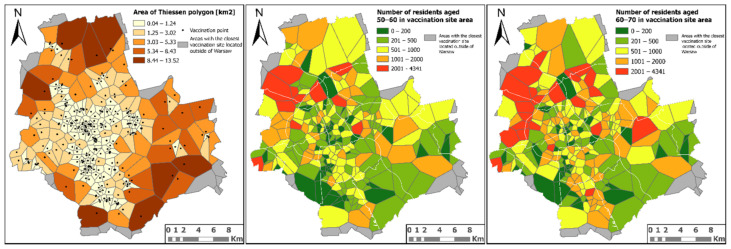
The results of the COVID-19 vaccination site availability analysis.

An analysis of the availability of vaccination sites using Thiessen polygons confirms the above results as well as indicates other areas where the availability of vaccination sites may be limited (Figure 10). The large diversity of the polygon surfaces indicates that the distribution of sites is ununiform. The larger the area covered by the range, the lower the availability of a given vaccination site. This is both about accessibility measured as the distance to be travelled to reach a given vaccination site and limited availability due to the large number of people living closest to a given site, which may translate into a long waiting time for vaccination. Such a situation occurs in two vaccination sites located in the Bielany district—*Centrum Medyczne Medycyna Rodzinna (LuxMed)* and *Przychodnia Conrada (SPZZLO Warszawa-Żoliborz*). As for the problem of a large number of people per vaccination site, apart from the previously indicated sites, the other affected sites are: one vaccination site located in the Żoliborz district, two in the Białołęka district and one each in the districts of Praga Północ, Praga Południe, Ursus and Wola (Figure 10). It should also be noted that the vaccination sites with highest numbers of people are the same sites for both analysed age groups, which means that in these areas the time needed to vaccinate people over 50 may be significantly extended.

### 3.3. Simulations of the Number of Days Needed to Vaccinate Each Studied Age Group

**Figure 11:**
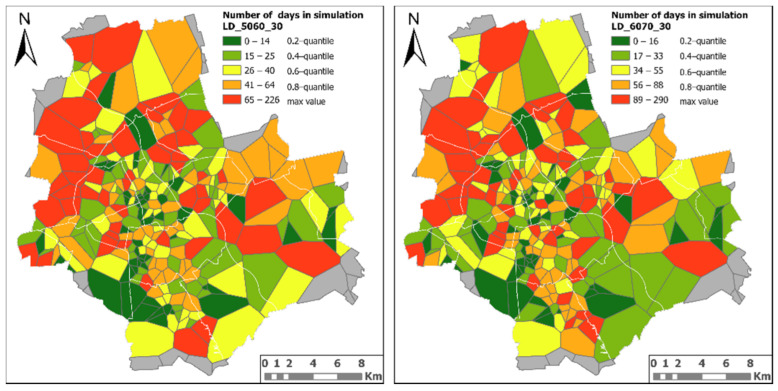
Number of days in simulations LD_5060_30, LD_6070_30.

This assumption is confirmed by the simulation in scenario 1, i.e., the number of days needed to vaccinate people in the analysed age groups, assuming an even distribution of vaccines between vaccination sites and the daily number of delivered doses equal to 30 (Figure 11). 

The analysis has shown a particularly disturbing phenomenon which is grouping of Thiessen polygons for which a long time is needed to vaccinate both age groups into larger areas. This may mean that the person has to go to a vaccination site a long way from home to be vaccinated. It should also be noted that in many cases the prolonged vaccination roll-out affects both studied age groups.

A solution to the problem of prolonged vaccination period may be the proposed method of optimizing the vaccine distribution between individual vaccination sites. This method allows to shorten the maximum number of days from 226 to 52 days in the LD_5060_OPTND simulation (Table 2). In the case of the 60–70 age group, the vaccination completion period in scenario 2 (LD_6070_OPTND) would be a maximum of 66 days, which means it was almost 4.5 times shorter than the even distribution of vaccines (LD_6070_30). Applying the method of optimizing the distribution of vaccines would allow the completion of vaccination in the 50–60 age group within 60 days at all vaccination sites, while in the case of even distribution it would be possible in 80% of vaccination sites. For the 60–70 age group, 80% of vaccination sites would complete the vaccination process after 88 days in the case of even distribution, for optimized distribution this time would be almost one month shorter and would be 59 days.

**Figure 12:**
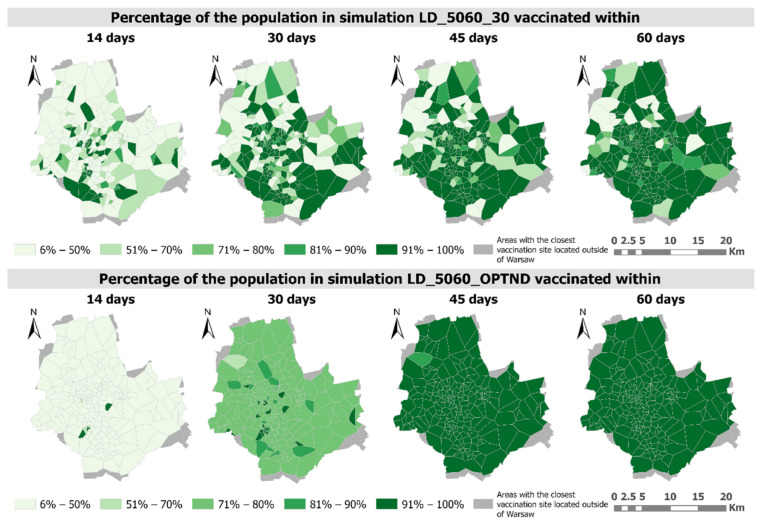
Percentage of the population of studied age groups that will be vaccinated within a specified number of days in LD_5060_30 and LD_5060_OPTND simulations.

**Figure 13:**
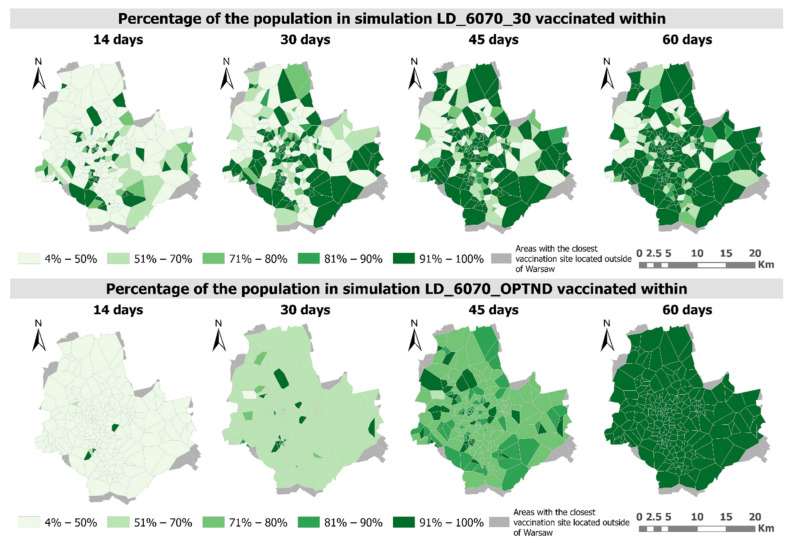
Percentage of the population of studied age groups that will be vaccinated within a specified number of days in LD_6070_30 and LD_6070_OPTND simulations.

Analysing the progress of the vaccination process measured by the percentage of people vaccinated, it is worth noting that with the proposed method of optimizing distribution, the vaccination process progresses more evenly (Figure 12 and Figure 13). 

For the 50–60 age group, after a period of vaccination of 45 days almost all people would have been vaccinated, while in LD_5060_30 simulation at some vaccination sites the percentage of the vaccinated would be below 50 (Figure 12). It is also worth noting that the vaccination rate is much higher in the optimized scenario (LD_5060_OPTND).

In the case of the 60–70 age group, due to the fact that it is characterized by a greater number of people, the percentage of people vaccinated within a vaccination period of 14, 30, 45 and 60 days is lower than in the case of the previously described group (Figure 13). Similarly, to the 50–60 age group, the vaccination process in the LD_6070_OPTND simulation is much more even than in the LD_6070_30 simulation and can be completed in almost 100% within a vaccination period of 60 days. In the case of even distribution, the problem of a low percentage of vaccinated people (less than 50%) is exacerbated, after 60 days it still affects 26 vaccination sites (Figure 13).

**Figure 14:**
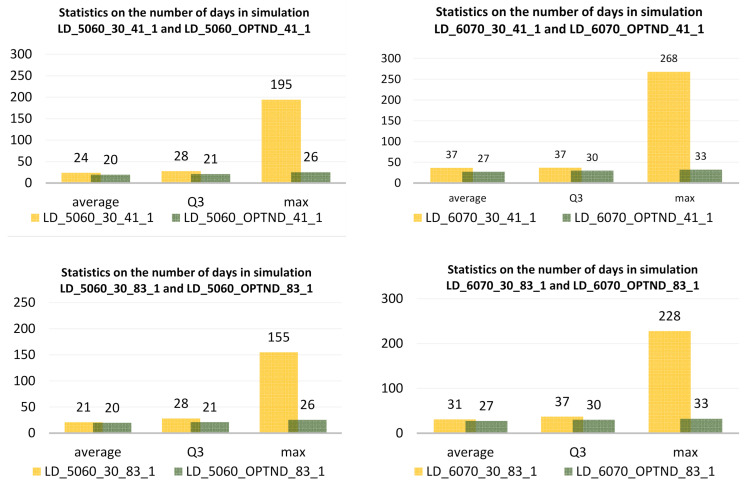
Statistics on the number of days at all vaccination sites in simulations LD_5060_30_41_1, LD_5060_OPTND_41_1, LD_6070_30_41_1, LD_6070_OPTND_41_1, LD_5060_30_83_1, LD_5060_OPTND_83_1, LD_6070_30_83_1, LD_6070_OPTND_83_1.

The proposed method of optimizing the distribution also enables a significant reduction in the time period needed to vaccinate all residents in the studied age groups with the first dose of the vaccine, in the case of vaccines requiring two doses (Figure 14). It is also worth noting that the time period required to vaccinate all people in a given age group with the first dose for the two-dose vaccines is shorter for the products with the longer interval required, in particular with the AstraZeneca vaccine. This is important because the first dose provides already a certain level of immunity to Sars-Cov-2 infection. On the other hand, the extended waiting time for the second dose increases the risk of contact with the virus, which, despite some acquired immunity, may result in infection.

Using the proposed method of optimizing vaccine distribution, the maximum number of days needed to vaccinate all people aged 50–60 years with the first dose of Pfizer/BioNTech and Moderna preparations would be 26 days and for the age group 60–70 it would be 33 days.

**Figure 15:**
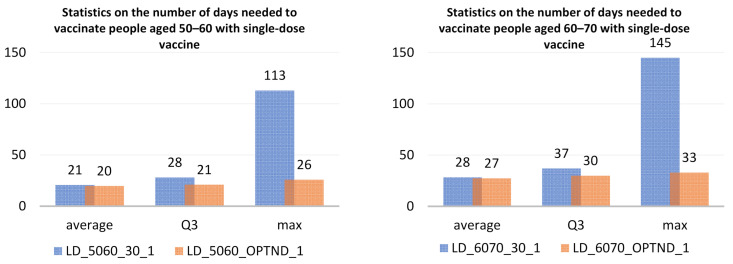
Statistics on the number of days at all vaccination sites in the simulations LD_5060_30_1, LD_5060_OPTND_1, LD_6070_30_1, LD_6070_OPTND_1.

A significant reduction in the length of the vaccination period regardless of age group can also be achieved with a single-dose vaccine currently produced by Johnson & Johnson. The simulations carried out in scenarios 3a and 3b showed that for the 50–60 age group the vaccination period would be a maximum of 113 days for even distribution and shorter by more than 4 times for optimized distribution (Figure 15). 

For the 60–70 age group, the maximum period for completion of vaccination, assuming optimized distribution, would be 33 days, which gives a total of 59 days needed to vaccinate all people aged 50–70 in Warsaw.

## 4. Discussion

In this study, a relatively simple method of optimizing vaccine distribution to each vaccination site was proposed and tested, based on the example of the capital city of Warsaw. This method is repeatable so that it can be successfully applied in other cities or local government units. There is no restriction in the level of territorial division here, it can be a district, voivodeship (province), as well as the entire country. If there is a need for an analysis of another type of administrative area, e.g., medical districts, it can also be implemented quickly. Thanks to the automation of the entire procedure, the information needed to manage and support decision-making regarding the supply of vaccines to each vaccination site can be obtained quickly, practically in the operational mode. Parameterization related to local circumstances is also possible, e.g., the maximum number of people vaccinated per day or the availability of vaccines of different companies. It is also possible to use various source data, but the key factor here is data on the place of residence of people in a specific age group, possibly from risk groups.

The use of data from the PESEL register, which was the primary source of data on inhabitants in a specific age group in this study, is associated with certain limitations and a likely slight underestimation of the number of inhabitants, and thus also the number of days needed to vaccinate the studied age groups. Considering the greater mobility of people under 50 years of age, repeating the analysis for the subsequent age groups to be vaccinated according to the vaccination program could lead to a significant underestimation and lack of reliability of the obtained results. There are other databases in Poland that could be used as a source of data for this task. Unfortunately, access to them is limited or they are not aggregated at the national level, which makes them difficult to use. Such data base could be the data stored within the primary health care system (POZ). The system includes local health care facilities providing medical care to all insured persons near their place of residence. In order to receive primary health care services patients, need to register by filling out a declaration and submitting it to a POZ clinic. From the declaration, a clinic collects a certain amount of information about a person, including some key data in the context of the presented analysis, i.e., PESEL number, which contains information about the date of birth and the address of residence. Due to the large dispersion of these data, their use would have to be preceded by their harmonization. It is also important to be aware of the fact that many people do not use public health care services, which means that the data obtained in this manner could still lead to some degree of underestimation. It is true that they could be supplemented with data from private clinics or individual medical practitioners, but it would require a long and labour-intensive harmonization phase.

The central data base that collects information about patients is the website of the Ministry of Health—pacjent.gov.pl. It contains information on all citizens having a PESEL number. Unfortunately, the address data in this data base are also collected from the PESEL register, so it provides information on a person’s registered address, not the residence address. It can be updated by the person, but only after logging in. Until 5 January 2021, only 4.7 million people in Poland [56] did so, so the use of these data would not significantly improve the results compared to those obtained based on the data from the PESEL register used in this study.

Another type of data could be commercial data, for example from mobile operators or banks. They could be particularly useful in the case of people under the age of 50, as the vast majority of them have a bank account or use a mobile phone. Both banks and mobile operators collect the date of birth or PESEL number of their clients and their address of residence. While in the case of banks, address data are rarely updated by their customers, in the case of data from mobile network operators, their data base could be supplemented with network login data and on this basis the residence address of a given person could be determined. Unfortunately, in the case of these two sources, there are also limitations resulting, for example, from their dispersion and the need to process them in order to obtain the right information.

Apart from access to data, a significant limitation of the conducted analysis is the lack of information on the details of the vaccination process at individual vaccination sites. This includes such information as: the hours and days of the week when a given vaccination site is open, the maximum number of vaccines that can be administered during a day, the availability of vaccine storage facilities, as well as the vaccines delivery schedule. With these data the period of time needed for vaccination of specific population groups or a specific percentage of the population could be determined more precisely. Another vital piece of data to increase the accuracy of the analysis is the information whether vaccines from a single manufacturer or several manufacturers are administered at a given vaccination site. Such information, in fact, would change the calculation of the number of days needed to vaccinate population age groups in the analysed scenarios.

The proposed analyses could be added more detail, taking into account the information on the transport accessibility of a given vaccination site, understood as the distance and time needed to reach it by foot, car or public transport. This is especially important in the context of limiting social contacts, and thus the risk of contagion. The best solution is the shortest possible route to get to the vaccination site or using your own car transport. To achieve this goal, it would be necessary to use network analyses that can also be performed with the use of GIS technology. It is a method that requires proper preparation of data on the roads and footpaths network, and its application to a large area requires a provision of large computing resources. Nevertheless, the method proposed in this study using Voronoi polygonization is a good approximation of the optimal “regionalization” for a particular vaccination site. And its advantage over network analyses are the lower computational requirements and the lack of need to have data on roads and footpaths network, which is usually time-consuming and expensive to obtain. However, when using Thiessen tessellation, it is worth being aware of its limitations. One of them is the lack of consideration of spatial barriers that may exist in cities or other analysed areas and significantly extend the route to a given vaccination site. These can be both natural barriers, such as a river, or anthropogenic barriers, such as a fenced estate. The solution to this problem could be to divide a city into sub-areas with pre-defined spatial barriers used as the dividing lines. Then the Thiessen tessellation would be performed on points within such sub-areas.

The vaccination scenarios analysed in the study consider two different approaches. The first was to distribute the same number of vaccines to each vaccination site and this was the scenario actually implemented in Poland at the initial phase of the vaccination program. In the following weeks, the national procurement system was launched in which each vaccination site declared its demand for vaccine supply for the subsequent week. However, this system has not been a platform for optimizing vaccine delivery in terms of population density or analysing where the number of vaccination sites should be increased to speed up the process. The approach we propose takes into account the number of inhabitants per vaccination site. As the analysis shows, this scenario has the advantage over the first one in that a greater percentage of the population in the city is vaccinated than in the first scenario (Figure 12 and Figure 13), within practically the same time. This is especially important in the case of airborne infectious diseases. In addition, hard lockdown of the city could be shortened in this case, which will make it less harsh for society and the economy. It should be emphasized that the proposed approach is not an alternative to the operation of the national COVID-19 vaccine procurement system but may support it by providing additional information and detecting deficiencies in the system. By using information on the place of residence of persons registered for vaccination, a plan of vaccine distribution to individual sites could be developed to minimize the time needed to vaccinate them.

**Figure 16:**
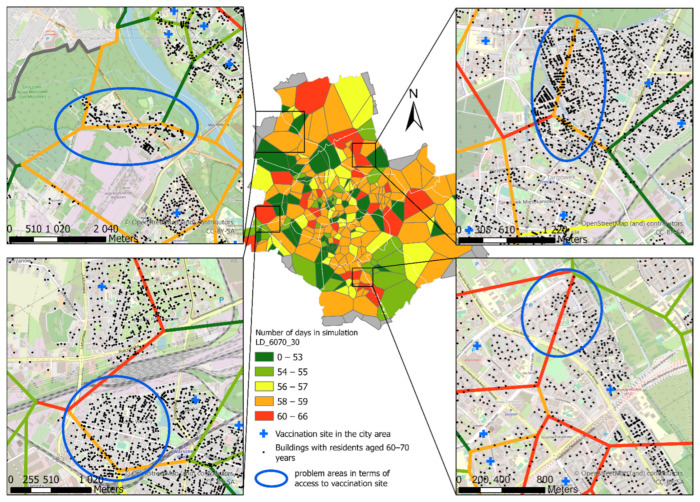
Example of identified areas characterised by low availability of COVID-19 vaccination sites for city residents aged 50–70.

A natural recommendation in the context of reducing the time needed to complete vaccination against COVID-19 is the launch of additional, ad hoc vaccination sites, including targeting mobile vaccination sites to some locations. The results obtained in the presented study can also be used for this purpose. The number of people per vaccination site and the availability of vaccines have a direct impact on the time required to vaccinate a given population. New sites should be located in areas with a large number of inhabitants in the analysed age groups or a large distance from the existing sites. To identify such areas information on the location of buildings inhabited by people of a given age group can be used. In places where their number is high and the distance to the nearest vaccination site is long, new COVID-19 vaccination sites should be established. An example of identifying such locations for the capital city of Warsaw is shown in Figure 16.

Locations with no vaccination sites are visible, and the number of buildings (Figure 16) and the number of inhabitants (Figure 9) are significant. The best solution in this type of situation seems to be directing mobile COVID-19 vaccination sites there. As a result, the need for residents to travel to distant vaccination sites will be reduced, as well as the risk of infection. Taking into account the needs of the economy, it is also possible to launch vaccination sites on the premises of industrial plants or university campuses which offer conditions for launching drive thru vaccination sites.

In the case of the capital city of Warsaw, with the current number of vaccination sites (and the assumptions adopted in this study), it is possible to vaccinate the entire population aged 50–70 with one dose of Pfizer/BioNTech and Moderna within 59 days, and vaccinate it with two doses within 118 days. With the single dose vaccine produced by Johnson & Johnson it would have been 59 days. In order for the analysis to be completely realistic, the schedules of delivery of vaccines of different manufacturers to Warsaw should be taken into account. Then it would actually be possible to estimate the time required to vaccinate a given population. Unfortunately, this type of data is not publicly available, hence it was not possible to use it.

In order to vaccinate 70% of the adult population of Warsaw with two-dose vaccines, 50 to 70 days (depending on the availability of vaccines) are currently needed (as of 1 April 2021) in order to vaccinate 565,549 people with the second dose and from 160 to 200 days to vaccinate the rest of adult people (875,000) with two doses. This means that the required level of herd immunity will be achieved after at least 210 days. It is possible to accelerate this process only by increasing the number of vaccination sites and extending their working hours, which, however, requires an increase in the number of medical personnel capable of administering vaccines. The faster immunisation of the population will certainly be facilitated by the approval of a single-dose vaccine from Johnson & Johnson in Europe. Then, the time required to vaccinate a significant part of the population to achieve an appropriate level of herd immunity will be significantly reduced.

While optimizing vaccine distribution may seem like a trivial task, it is so only in the case of a single dose vaccination, when the number of vaccination sites and the supply of vaccines are sufficient to ensure the continuity of the vaccination process. In a situation where multiple doses of vaccine are required, as well as special storage conditions, there are suspensions or delays in the delivery of vaccine doses, and in addition, the number of cases increases, this is quite complicated. Then, the dynamics of COVID-19 incidence in a given area should also be taken into account in the context of population density, mobility of inhabitants, i.e., elements that affect the probability of airborne infection spread.

## 5. Conclusions

The proposed method of analysing and optimizing the distribution of COVID-19 vaccines to particular vaccination sites is based on the Thiessen tessellation and takes into account the population density in a specific age group. The aim of this approach was to minimize the time needed to vaccinate the indicated population group and to limit the mobility of inhabitants. This solution makes it possible to obtain quick information on the availability of each vaccination site, their workload, the required number of vaccines to meet the vaccination needs or the number of vaccines needed to vaccinate a specific part of the population. In addition, it enables identification of areas with deficiencies in the vaccination sites network and it would be required to activate additional sites or to locate ad hoc or mobile vaccination sites. This information could complement the national vaccination system and support the management of vaccine distribution in each area and the entire country. The scenario taking into account the population density allows the vaccination time to be shortened even several times (depending on the availability of vaccines).

Observing the current situation in the world, it can be seen the predictions of Skegg et al., who stated that:

*A nationalistic rather than a global approach to COVID-19 vaccine availability, distribution, and delivery will make a pessimistic outcome much more likely. Additionally, unless countries work together to scale up prevention efforts, the risk of other pandemics, or other transboundary disasters with similar consequences, including those fuelled by climate change, will remain a constant threat* [57].

It is hard to disagree, but especially at the height of the pandemic, the national interest will be a priority for the authorities of each countries. However, unless all countries in the world have access to COVID-19 vaccines, it will be virtually impossible to completely eradicate the pandemic.

## Figures and Tables

**Table 1:** Description of the simulations conducted under the three scenarios.

Scenario	Simulation Abbreviation	Description of the Simulation
1	LD_5060_30	Number of days needed to vaccinate people aged 50–60 years, based on 30 doses per day (same number of doses at each vaccination site) and any interval between doses
1	LD_6070_30	Number of days needed to vaccinate people aged 60–70 years, based on 30 doses per day (same number of doses at each vaccination site) and any interval between doses
1	LD_5060_30_41_1	Number of days needed to vaccinate people aged 50–60 years with the first dose based on 30 doses per day (same number of doses at each vaccination site) and an interval between doses of 41 days
1	LD_6070_30_41_1	Number of days needed to vaccinate people aged 60–70 years with the first dose based on 30 doses per day (same number of doses at each vaccination site) and an interval between doses of 41 days
1	LD_5060_30_83_1	Number of days needed to vaccinate people aged 50–60 years with the first dose based on 30 doses per day (same number of doses at each vaccination site) and an interval between doses of 83 days
1	LD_6070_30_83_1	Number of days needed to vaccinate people aged 60–70 years with the first dose based on 30 doses per day (same number of doses at each vaccination site) and an interval between doses of 83 days
3a	LD_5060_30_1	Number of days needed to vaccinate people aged 50–60 years with the single dose vaccine based on 30 doses per day (the same number of doses at each vaccination site)
3a	LD_6070_30_1	Number of days needed to vaccinate people aged 60–70 years with the single dose vaccine based on 30 doses per day (the same number of doses at each vaccination site)
2	LD_5060_OPTND	Number of days needed to vaccinate people aged 50–60 years, assuming an optimized number of doses per day and any interval between doses
2	LD_6070_OPTND	Number of days needed to vaccinate people aged 60–70 years, assuming an optimized number of doses per day and any interval between doses
2	LD_5060_OPTND_41_1	Number of days needed to vaccinate people aged 50–60 years with the first dose based on the optimized number of doses per day and an interval between doses of 41 days
2	LD_6070_OPTND_41_1	Number of days needed to vaccinate people aged 60–70 years with the first dose based on the optimized number of doses per day and an interval between doses of 41 days
2	LD_5060_OPTND_83_1	Number of days needed to vaccinate people aged 50–60 years with the first dose based on the optimized number of doses per day and an interval between doses of 83 days
2	LD_6070_OPTND_83_1	Number of days needed to vaccinate people aged 60–70 years with the first dose based on the optimized number of doses per day and an interval between doses of 83 days
3b	LD_5060_OPTND_1	Number of days needed to vaccinate people aged 50–60 years with the single dose vaccine assuming an optimized number of doses per day
3b	LD_6070_OPTND_1	Number of days needed to vaccinate people aged 60–70 years with the single dose vaccine assuming an optimized number of doses per day

**Table 2:** Values of selected quantiles for simulations carried out in scenario 2.

Simulation	0.2-Quantile	0.4-Quantile	0.6-Quantile	0.8-Quantile	Max Value
LD_5060_OPTND	39	40	41	42	52
LD_6070_OPTND	53	57	58	59	66

## Data Availability

Most of the data used in this study are publicly available. They are described in detail in the paper and there are links to them. Data from the PESEL register are available in an aggregated form at the request (https://www.gov.pl/web/cyfryzacja/udostepnianie-danych-z-rejestru-pesel1, accessed on 12 March 2021).
